# Optimized strategies for developing high-speed muscle activity monitors utilizing multi-resolution energy operator

**DOI:** 10.3389/fbioe.2025.1565987

**Published:** 2025-04-01

**Authors:** Shenglin Wang, Guosheng Zhao, Yiwei Liao

**Affiliations:** College of Computer Science and Information Engineering, Harbin Normal University, Harbin, China

**Keywords:** EMG activity monitor, change-point detection, Teager-Kaiser energy operator (TKEO), double-threshold detector, convolutional neural network (CNN)

## Abstract

**Introduction:** Electromyographic (EMG) activity monitoring constitutes the core of foundational research for the application of EMG signals in medical diagnostics, sports science, and human-machine interaction. However, the current research trend predominantly focuses on the recognition technologies of EMG signals, while the techniques for accurately detecting the onset and offset points of muscle activity—the change-point detection of EMG signals—have not received the necessary attention and thorough investigation.

**Methods:** A novel method for EMG signal activity detection based on a variant version of the Teager-Kaiser energy operator (TKEO), namely the multi-resolution energy operator (MTEO), is proposed. Two strategies for constructing EMG activity monitors using MTEO are presented. One is a threshold-based detector (MEOTD) relying on signal baseline segment information, and the other is a detector mimicking the structure of a convolutional neural network (MEONND) without requiring prior knowledge of the signal. A semi-subjective evaluation model based on the Analytic Hierarchy Process (AHP) is used to evaluate the performance of the monitors on real EMG data.

**Results and discussion:** The results show that the MTEO has stronger preprocessing ability for EMG signals, and that the MTEO-based monitors are more reliable and accurate. In particular, the MEONND can achieve both computational efficiency and accuracy simultaneously. The proposed method for EMG signal activity detection improves both detection quality and efficiency without increasing algorithm complexity. This method can be applied to various fields that involve EMG signal analysis, such as ergonomics, human-machine interaction, and biomedical engineering.

## 1 Introduction

Electromyography (EMG) signals contain abundant human motion information and are commonly used as a source of information for various applications, such as human-computer interaction (Chen et al., 2021; Chopra, 2021), rehabilitation robots (Yang et al., 2017), mechanical exoskeletons (Vigotsky et al., 2018; Wang et al., 2019), sports training (Abe et al., 2009), rehabilitation assessment (Zhao et al., 2018), medical diagnosis (Badani et al., 2017), etc. There are two main research directions for EMG signals: localization and recognition. Localization is the extraction of valid motion temporal information by pinpointing the onset and offset times of muscular activity. Recognition is the classification of specific movements to obtain human movement gestures. However, these two research directions are not mutually exclusive. For example, a combination of multiple location-based On-Off control detectors can also be used for action recognition (Tamura et al., 2010; Yamashita et al., 2012); alternatively, the EMG signal could be segmented into activity segments first and then perform classification only on the activity segments in order to enhance the accuracy of the classification algorithm and reduce the consumption of computational resources (Di Nardo et al., 2022). In this paper, we focus on the localization techniques for EMG signal activity detection.

The EMG signal model can be generally simplified as the superposition of noisy signal (baseline segment) and EMG signal (activity segment), so the EMG signal activity detection can be viewed as a source separation problem. Before deep learning was applied to source separation, the Time-Frequency Domain method based on Short-time Fourier Transform (STFT) was the main approach. However, STFT requires a longer window length to ensure a certain frequency domain resolution, which compromises its real-time performance. Deep learning detectors based on the time domain can address the real-time issue well, but they face the challenges of too many parameters and insufficient training data in the training stage. Especially, human bio-signals are very private and unique, and it is harder to obtain such labeled data. Therefore, even though the pattern recognition method represented by deep learning has good detection performance in experimental scenarios, such as recurrent neural networks (RNN) (Akef Khowailed and Abotabl, 2019) and long short-term memory (LSTM) (Ghislieri et al., 2021), it is still not ready for practical application until the robustness of Few-shot learning is solved. Recently published statistical methods–CUSUM (Jeske et al., 2009), Profile Likelihood Maximization (PLM) (Selvan et al., 2018), Sample Entropy (SE) (Zhang and Zhou, 2012), and Bayesian changepoint analysis methodology (Tenan et al., 2017) - are appealing alternatives. However, the performance of the monitor depends heavily on the prior knowledge of distribution assumption, probability density function selection, and parameter estimation. Therefore, statistical-based monitors may fail when dealing with different types of EMG signals (e.g., fast, slow, or maximum voluntary contraction) if a wrong prior assumption is chosen (Selvan et al., 2018).

Another common type of EMG activity monitor is the time-domain-based thresholding method, especially the double-threshold method. The threshold method does not require a large number of learning samples and only needs a certain length of baseline information for change-point detection of the signal. Because of its computational simplicity and real-time performance, it is preferred by relevant applications. However, it is also the single model structure that makes the threshold methods less robust to noise-containing signals and more sensitive to noise. Moreover, the fixed threshold factor worsens this drawback, so that threshold-based monitors are considered less reliable than those based on statistics or pattern recognition.

It has been shown that a simple but well-configured detector can match or even outperform many complex models in time-series tasks such as change-point detection, outlier detection, or anomaly detection (Elsayed et al., 2021). A prerequisite for a simple detector to work well is to pre-process the signal to obtain more distinctive signal features. Previous studies have shown that the Teager-Kaiser energy operator (TKEO) can effectively enhance the performance of EMG activity monitors, either by using the TKEO as a detector (Xiaoyan and Aruin, 2005; Li et al., 2007; Crotty et al., 2021) or as a signal conditioning technique (Solnik et al., 2008; 2010; Tenan et al., 2017; Kaur et al., 2018; Selvan et al., 2018; Bengacemi et al., 2020; Crotty et al., 2021). A key feature of the TKEO is that it can extract the time-frequency information of signals through an extremely narrow window containing only three samples (Chopra, 2021). Due to the simplicity of the TKEO operation, threshold detectors with the same low computational resource consumption properties are usually combined to ensure high real-time performance of the algorithms. However, the narrow ‘sight’ when processing the signal makes the TKEO very susceptible to noise (Agarwal and Gotman, 1999; Sa et al., 2002; Palmu et al., 2010; Wang et al., 2021; Hamilton and Chitrapu, 1995), and the use of threshold detectors aggravates this problem.

Actually, as a technique derived from speech signal processing, the TKEO has developed several variants to address the noise-sensitive problem. The aim of these variants is to reduce the false alarm probability and detection bias of energy operator methods by decreasing their sensitivity to noise, but this requires more computational resources. Agarwal and Gotman (Agarwal and Gotman, 1999) proposed an asymmetric variant of the TKEO that segments electroencephalogram signals into piece-wise stationary sections, based on Plotkin and Swamy‘s study (Plotkin and Swamy, 1992). Kamath (Kamath, 2012) constructed a nonlinear scatter plot for the TKEO of R-R interval series to detect congestive heart failure. Benalcazar-Parra et al. (Benalcazar-Parra et al., 2019) did not modify the TKEO, but they proposed a new TKEO-based model for intrauterine pressure (IUP) estimation that optimizes the clinical characteristics of IUP such as continuous pressure, maximum pressure and tension by surface electronic hysterogram (EHG), thus enabling non-invasive labor monitoring. Among these variant versions of the TKEO, the multi-resolution Teager-Kaiser energy operator (MTEO) has demonstrated good performance in applications such as glottal closure instants detection (Wu et al., 2017) and bearing fault detection (Wang et al., 2021; Xu et al., 2021). However, few studies have explored the use of MTEO for EMG activity detection (Wang et al., 2022).

This paper presents two strategies for constructing EMG activity monitors based on the MTEO–the traditional threshold detector (MEOTD) that heavily relies on baseline segment information, and the more robust and fast neural network-like detector (MEONND) that does not require any prior knowledge about the signal. In addition, a parameter tuning and performance evaluation model is constructed by the Analytic Hierarchy Process (AHP) to address the problem of overly subjective parameter selection. Compared to the state-of-the-art monitors, the effectiveness of the MEOTD and MEONND is highlighted.

The remainder of this paper is structured as follows. Section 2 provides a brief description of the background of the TKEO and MTEO. The details of the proposed monitors and the performance assessment method are described in Section 3, while we also present a model for parameter tuning. Section 4 verifies the improvement in signal conditioning performance of the MTEO compared to the TKEO and compares it with the state-of-the-art statistical-based and pattern recognition monitors to highlight the effectiveness of the MEOTD. Furthermore, by analyzing how the monitor performance varies with the parameters, we attempt to explain the reasons for the performance improvement. Finally, Section 5 draws some conclusions.

## 2 Background of the TKEO and MTEO

In general, signal processing (e.g., speech signals, physiological signals) is often simplified into a linear filter system, but EMG is actually a non-linear signal. Teager revealed that the generation of a speech signal is a non-linear process (Teager and Teager, 1990). Following Teager’s work, Kaiser proposed a simple operator to calculate the instantaneous “energy” of the signal and at the same time, the nonlinearity of the signal is taken into account (Kaiser, 1990).

According to Simple Harmonic Motion (SHM), the total energy of the system can be expressed as the sum of kinetic energy and potential energy (Equation 1):

$$E=\frac{1}{2}kx^{2}+\frac{1}{2}mx^{2}$$ (1)

where k represents the spring constant and m represents the motion of a mass. According to the law of conservation of mechanical energy, we can obtain Equation 2:

$$E=\frac{1}{2}kA^{2}$$ (2)

The symbol A comes from the general solution of the simple harmonic motion given by $x\left(t\right)=\mathrm{Acos}\left(\omega t+\phi \right)$ where A is the amplitude, ϕ is the phase angle and ω is the angular frequency. For a given spring oscillator, the angular frequency is only related to its spring constant and mass, and the formula is as Equation 3:

$$\omega =\sqrt{\frac{k}{m}}\Rightarrow k=\omega^{2}m$$ (3)

substituting for k and solving, we obtain:

$$E=\frac{1}{2}\omega^{2}mA^{2}$$ (4)

Therefore, as shown in Formula 4, the energy E is proportional to $\omega^{2}$ and $A^{2}$ , which can be expressed as Equation 5:

$$E\propto \omega^{2}A^{2}$$ (5)

Thus the energy of a signal is proportional not only to the square of the amplitude but also to the square of the frequency (Kaiser, 1990). Subsequently, Kaiser gave the relationship between energy and discrete sampling points (Equation 6):

$$E_{n}=x_{n}^{2}-x_{n+1}x_{n-1}=A^{2}\sin^{2}\left(\omega \right)\approx \omega^{2}A^{2}$$ (6)

Where $E_{n}$ is the output of the algorithm and represents the energy of the n th sample point, and $x_{n}$ is the value of the current sample point. Note that a more general representation of $E_{n}$ is $\Psi \left[x\left(n\right)\right]$ , that is, $\Psi \left[x\left(n\right)\right]=x_{n}^{2}-x_{n+1}x_{n-1}$ is the most common formula for the TKEO in the discrete domain. Kaiser explained the relationship shown in Equation 6 by measuring the ‘energy’ of the signal by the square of the frequency and the square of the amplitude. Furthermore, some researchers have also interpreted $\omega^{2}A^{2}$ as a frequency-weighted energy (FWE) (Agarwal et al., 1998; O’Toole et al., 2014). From this point of view, the signal ‘energy’ obtained by the TKEO is not only low weighted for low frequency, but also high weighted for high frequency. This makes the TKEO an ideal method of signal conditioning, as it reduces the background noise while strengthening active segment signals, which facilitates a more accurate determination of EMG change-point. In fact, due to the above-mentioned properties, the noise present in the baseline segment is also amplified, making the TKEO very sensitive to noise (Agarwal and Gotman, 1999; Sa et al., 2002; Palmu et al., 2010; Wang et al., 2021). Currently, several modified versions of the TKEO have been proposed to address the noise sensitivity problem. Actually, the TKEO ontology can be viewed as a special case in a more general framework of energy operators. This provides a theoretical basis for the improvement of the TKEO.

Kumaresan et al. generalized the TKEO into a matrix framework and interpreted this general framework through the determinant of a Toplitz matrix containing the signal and its derivatives (Kumaresan et al., 1992), and obtained the determinant of the signal in the discrete domain of Equation 6 as Equation 7:

$$\Psi_{d}\left[x\left(n\right)\right]=\left(\begin{array}{cc} x\left[n\right] & x\left[n-1\right]\\ x\left[n+1\right] & x\left[n\right] \end{array}\right)$$ (7)

The determinant is time-invariant for a signal with constant frequency. If such matrix is generalized to an M×M Toeplitz matrix by adding delayed $x\left[n\right]$ up to $x\left[n\pm \left(M-1\right)\right]$ , the determinant is also time-invariant but for signals with multi frequency (Kumaresan et al., 1992; Boudraa and Salzenstein, 2018):

$$\Psi_{M}\left[x\left(n\right)\right]=\left(\begin{array}{cc} x\left[n\right] & x\left[n-\left(M-1\right)\right]\\ x\left[n+\left(M-1\right)\right] & x\left[n\right] \end{array}\right)$$ (8)

As shown in Equation 8, it is a feasible improvement idea to enhance the conditioning performance of the energy operator on the signal by taking different values of M to strengthen different frequency components of the signal and then fusing the enhanced sub-signals by some method.

Based on the above theoretical basis, Choi (Choi and Kim, 2002; Choi et al., 2006) et al. proposed a Multiresolution Teager energy operator (MTEO). Firstly, MTEO obtains the k -TEO of the corresponding sub-signals by taking multiple M values; then, a maximum pooling technique is used to fuse the k -TEO of the sub-signals, thus, improving the sensitivity of the algorithm to different frequencies of the action potentials and maximizing the difference between the baseline signals and the active segment signals. The details are as follows:

$$\Psi_{k}\left[x\left(n\right)\right]=x^{2}\left(n\right)-x\left(n+k\right)x\left(n-k\right)$$ (9)

$$p\left(n\right)=\max\left(\Psi_{1}\left[x\left(n\right)\right],\Psi_{2}\left[x\left(n\right)\right],\ldots ,\Psi_{k}\left[x\left(n\right)\right]\right).$$ (10)

Specifically, Equation 9 is obtained by replacing M−1 in Equation 8 with k . Setting various scale factors k in Equation 9 can decompose multiple k -TEO corresponding to different frequencies, and then all k -TEO are aggregated by Equation 10 to obtain the final ‘energy’ of the signal. In Equation 9, the scale parameter k is a positive integer, measuring the time distance between $x\left(n\pm k\right)$ and $x\left(n\right)$ . A larger k indicates a larger time distance between the involved samples and the relevance between the samples decreases. Equation 10 pools the sub-signals obtained from Equation 9 through a pooling layer structure similar to the common neural network algorithms, covering more local samples, so theoretically, the MTEO can achieve stronger robustness and adaptability.

In addition, according to previous studies (Palmu et al., 2010; O’Toole et al., 2014; Wu et al., 2017) and the experimental verification in this paper, full-wave rectification of the TKEO can improve the detection performance of the monitor. The rectified TKEO is defined as Equation 11:

$$\left|\Psi \right.\left[x\left(n\right)\right]\left|=\right.\left|x^{2}\left(n\right)-x\left(n+1\right)x\left(n-1\right)\right|$$ (11)

But at present, and to the author’s knowledge, whether rectification can improve the performance of the MTEO has not been experimentally verified. Therefore, this paper will conduct experimental verification on this issue. The rectified MTEO is defined as Equation 12:

$$\left|p\right.\left(n\right)\left|=\max\right.\left(\left|\Psi_{1}\left[x\left(n\right)\right]\left|,\ldots ,\right.\left|\Psi_{k}\left[x\left(n\right)\right]\right|\right.\right)$$ (12)

For briefness, the rectified TKEO defined above is denoted as the aTKEO and the rectified MTEO is denoted as aMTEO thereafter (*a* is short for absolute). This paper will use the conditioning performance of the TKEO on EMG signals as a reference to quantitatively analyze the performance of the MTEO. At the same time, the effectiveness of the proposed method is verified by comparing the state-of-the-art methods.

## 3 Materials and methods

### 3.1 EMG acquisition

**FIGURE 1:**
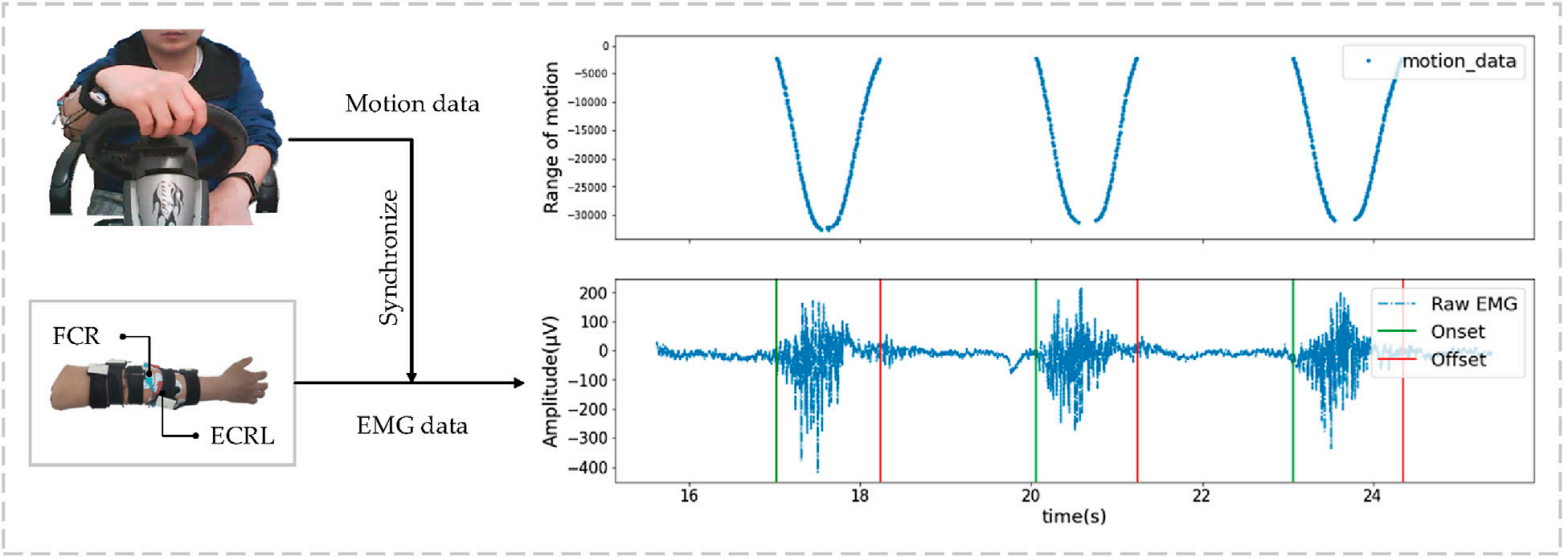
The signal acquisition method and the raw EMG data with labels.

To obtain real EMG signals with labels, similar to the method implemented by Chopra et al. (Chopra, 2021), this paper uses a high-precision gaming steering wheel to locate the change-points of EMG signals. Specifically, the experimental acquisition device was a smart wearable recorder, ErgoLAB (ErgoLAB, 2025), with a sampling frequency of 1,024 Hz, and it was enabled with only 50 Hz IDF filtering. The A/D converter chip of the gaming steering wheel provided 16-bit resolution analog quantities, which could acquire values between 0 and ±32,767, with positive and negative values representing the quantization of the steering wheel’s positive and negative motion amplitudes, respectively. Following approval by the local university research ethics committee, twenty healthy right-handed participants (10 males and 10 females, aged 26.5 ± 3.6) participated in the experiment, completing one signal acquisition per day from August 6 to 12, 2019, for a total duration of 6 days. During each signal acquisition, electrodes were attached to the extensor carpi radialis longus (ECRL) and flexor carpi radialis (FCR) of the participants’ right forearms after skin treatment (positive and negative electrode pairs were placed 1 cm apart at the center of the muscle belly, and the reference electrode was placed on the processus spinosus with a 1.8 cm diameter electrode sheet). Participants operated the gaming steering wheel to the left and right 30 times in the most natural state, respectively, without limiting speed or force, but they were asked to avoid additional movements. The control signals collected by the gaming steering wheel can be considered as human motion data, and the motion data were synchronized with EMG data by time stamping to obtain relatively accurate Onset and Offset labels. The raw data and the acquisition device are shown in Figure 1.

It should be noted that, due to the antagonistic characteristics of the muscles, the active muscles (spontaneous muscles) of the right forearm are different when the steering wheel is operated to the left or right, which results in half of the data acquired not being accurately detected at the change-points of the active segments and needing to be discarded. Therefore, a total of 7,200 active segments with onset and offset labels that could be used for change-point detection were obtained in the experiment.

### 3.2 Data processing

As a powerful pre-conditioning technology, the energy operator can replace many computationally complex and time-consuming conventional pre-processing methods (e.g., Butterworth filters, Baseline detrending, etc.). However, the energy operator is very sensitive to signal noise. This is because, as proposed by Vakman et al. (Vakman, 1996), when extracting the amplitude and frequency components of the signal with the energy operator, the amplitude $a\left(t\right)$ and frequency $\omega \left(t\right)$ represented by the energy operator are related to the signal as follows:

$$a\left(t\right)=\frac{\Psi \left(u\right)}{\sqrt{\Psi \left(u^{\prime }\right)}}$$ (13)

$$\omega \left(t\right)=\sqrt{\frac{\Psi \left(u^{\prime }\right)}{\Psi \left(u\right)}}$$ (14)

where u is the signal. In view of (Equations 13, 14), if $\Psi \left(u^{\prime }\right)\to 0$ and $\Psi \left(u\right)\to 1$ , then a condition occurs where the amplitude tends to infinite while the frequency tends to zero. This phenomenon leads to spikes in the signal processed by the energy operator that are difficult to eliminate and affect the performance of the detector, which is hardly expectable. Due to the large amplitude of the pulse spike, the traditional sliding average filtering method cannot eliminate the influence. In this paper, order statistics is used to solve this issue (Bengacemi et al., 2020). Specifically, a median filter is used instead of the traditional mean filter. The energy value $E_{j}$ of the j th frame of the median filter is expressed as Equation 15:

$$E_{j}=median\left(\left\{\right|\Psi \left(n\right)|,\left.\left(j-1\right)L+1\leq n\leq Lj\right\}\right)$$ (15)

**FIGURE 2:**
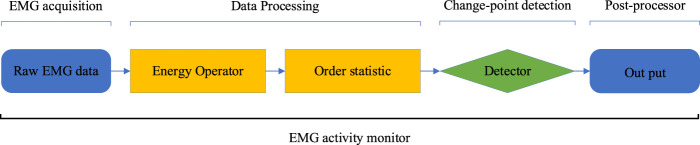
The construction strategy of the EMG activity monitor.

The parameter L here represents the window length for executing the order statistic. A detailed analysis of this parameter is presented in Section 3.5. The above signal processing techniques can effectively improve the robustness of the energy operator for EMG activity monitoring. Figure 2 illustrates the construction strategy of the EMG activity monitor in this paper.

### 3.3 Detector

In general, detectors combined with the TKEO are threshold methods. An important reason is the simple structure and low complexity of the threshold-based detectors, which benefit them from low detection latency (Yang et al., 2017). Since the effectiveness of the MTEO for EMG activity monitoring has not been deeply developed, this paper follows this idea of structural simplicity and low complexity, and explores the traditional threshold-based detector (MEOTD), as well as an alternative modified version (MEONND).

#### 3.3.1 Double threshold detector

The effective operation of the threshold method is based on the assumption that the noise variance (i.e., the signal baseline) is *a priori* known. Therefore, before change-points detection, it is necessary to ensure that the initial signal recording represents only noise (no activity) so that it can be used for the noise level estimation, which is a prerequisite for the standard threshold method. Signal feature extraction is performed in the baseline segment through a sliding window. The details are as follows: the selected baseline segment $L_{b}$ is divided into frames with $L_{s}$ window length and s step size, then, the mean value $M_{s}^{th}$ and standard deviation ${Std}_{s}^{th}$ of each frame are calculated. Finally, the mean of all $M_{s}^{th}$ and ${Std}_{s}^{th}$ is used to obtain the final baseline segment information. Thus, the first threshold in this paper can be formulated as Equation 16:

$$Th_{1}=\frac{1}{\left\lfloor Win\_num\right\rfloor }\sum_{th=0}^{\left\lfloor Win\_num\right\rfloor }M_{s}^{th}+j*\frac{1}{\left\lfloor Win\_num\right\rfloor }\sum_{th=0}^{\left\lfloor Win\_num\right\rfloor }{Std}_{s}^{th}$$ (16)

The parameter j here represents the threshold scaling factor which allows us to control the tradeoff between false alarms and detection probabilities. j is an important parameter and a detailed analysis is presented in Section 4. Win_num refers to the number of sampling windows, each of which represents a certain number of samples in the EMG signal.

**FIGURE 3:**
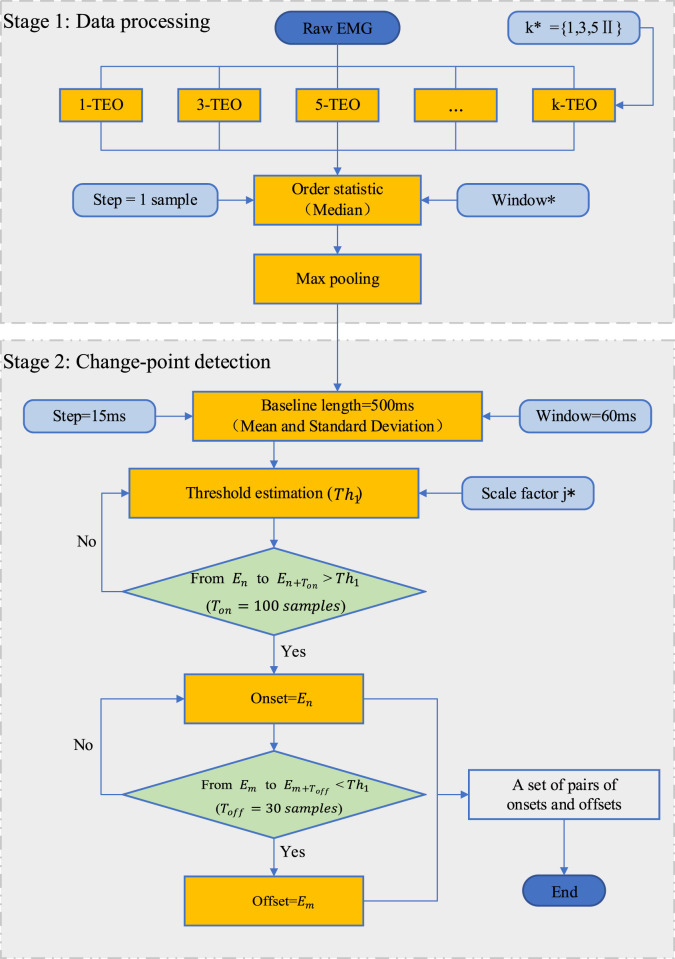
The framework of MEOTD. The round angle rectangles represent the algorithm parameters, where the parameters marked with an asterisk need to be tuned.

A prominent advantage of the double-threshold method in EMG activity monitoring is that the threshold parameters can be set according to the physiological characteristics of the muscle activity, thus avoiding secondary threshold estimates that generate more parameter settings. There are two characteristics of muscle contraction: firstly, there is a minimum contraction duration from activation to rest (typically, ≥100 m); secondly, there is a minimum contraction switching time after muscle rest to the next muscle activation (typically, 25–30 m) (Yang et al., 2015; Ghislieri et al., 2021). Therefore, a threshold $T_{on}$ can be set for onset point determination based on the first feature of the muscle contraction; and a threshold $T_{off}$ for offset point according to the second. For each new analysis sampling point, apply $T_{on}$ and $T_{off}$ to decide on the activity status of the considered sampling point according to Equation 17:

$$\left\{\begin{array}{l} If\;\left[E_{n}:E_{n+T_{on}}\right]\rangle Th_{1},{\;E}_{n}\;presents\;an\;EMG\;activity\\ If\;\left[E_{m}:E_{m+T_{off}}\right]<Th_{1}\;after{\;E}_{n},{\;E}_{m}\;presents\;no\;EMG\;activity\\ else:No\;events\;detected \end{array}\right.$$ (17)

where $E_{n}$ and $E_{m}$ represent the onset and offset of the active segment identified by the EMG activity monitor, respectively, and [x: y] denotes a continuous sampling segment from x to y. By appropriately setting $T_{on}$ and $T_{off}$ , the false samples can be effectively eliminated (Rashid et al., 2019). The framework of MEOTD is shown in Figure 3.

#### 3.3.2 Neural network-like detector

**FIGURE 4:**
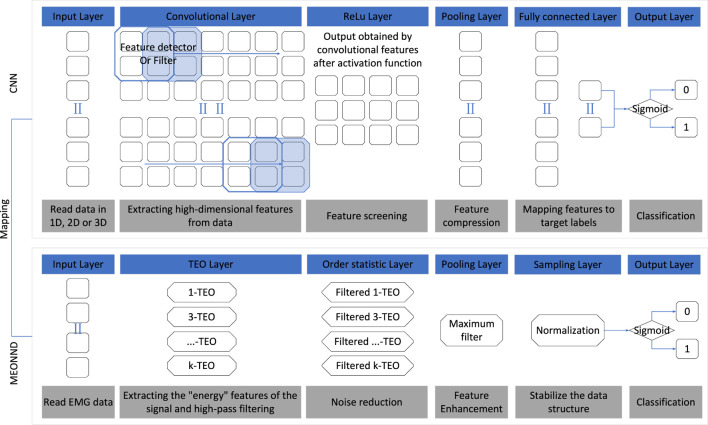
Mapping relationship of model layout between CNN and MEONND. The blue blocks represent the names of the functional components and the gray blocks introduce the functions of the corresponding components.

First, it is important to state that convolutional neural network (CNN) models have powerful feature extraction and generalization capabilities, which are the core advantages of neural network algorithms. The feature extraction and generalization capabilities of CNN models mainly stem from the key steps such as the design of convolutional kernels, the training of hyperparameters, and the application of regularization techniques (Zhang et al., 2017). The method proposed in this paper fixes the neuron parameters, making the model lose the adaptive learning ability for signal features. Whether the fixed-parameter neural network algorithm still falls into the category of neural networks is controversial, but it still simulates information processing based on neurons and weighted connections, and can also express the complex properties of nonlinear functions. Therefore, this paper refers to it as a neural network-like algorithm. The proposed neural network-like algorithm transformed by the MTEO method is named MEONND (Multi-resolution Energy Operator with Neural Network Architecture Detector), and its mapping relationship with the convolutional neural network algorithm framework is shown in Figure 4.

Figure 4 shows the model layout and component functions. The design concept of MEONND is based on the assumption that a trained neural network is capable of performing the change-point monitoring tasks. Therefore, by replacing the components that need to be trained in a reasonable manner and keeping the components that do not need to be updated, a detector similar to the trained neural network can be constructed in theory. Since the parameters are set in advance and the neural network model is no longer a black-box, the fixed-parameter neural network offers simplicity and interpretability over the traditional neural network, and the parameters are tuned quickly and do not depend on the amount of training data, which enables more robust algorithm design for small data sets.

As shown in Figure 4, MEONND follows the same architecture of the basic CNN algorithm, and the replaced components are also functionally very similar to the components before being replaced. Specifically, the convolutional layer and the activation layer controlling the output of the convolutional layer in the CNN are substituted with multiple TEOs, each with different k and an order statistics component. Since the traditional TKEO can be expressed by the following neuronal relational (Equation 18) (Akef Khowailed and Abotabl, 2019):

$$C\left(n\right)=W_{1}{\left(w_{11}x\left(n+1\right)\right)}^{2}+W_{2}{\left(w_{22}x\left(n\right)\right)}^{2}+W_{3}{\left(w_{33}x\left(n-1\right)\right)}^{2}+W_{4}{\left(w_{14}x\left(n+1\right)+w_{34}x\left(n-1\right)\right)}^{2}$$ (18)

If TKEO is updated to MTEO, Equation 18 can be rewritten as:

$$C\left(k\right)=W_{1}{\left(w_{11}x\left(n+k\right)\right)}^{2}+W_{2}{\left(w_{22}x\left(n\right)\right)}^{2}+W_{3}{\left(w_{33}x\left(n-k\right)\right)}^{2}+W_{4}{\left(w_{14}x\left(n+k\right)+w_{34}x\left(n-k\right)\right)}^{2}$$ (19)

**FIGURE 5:**
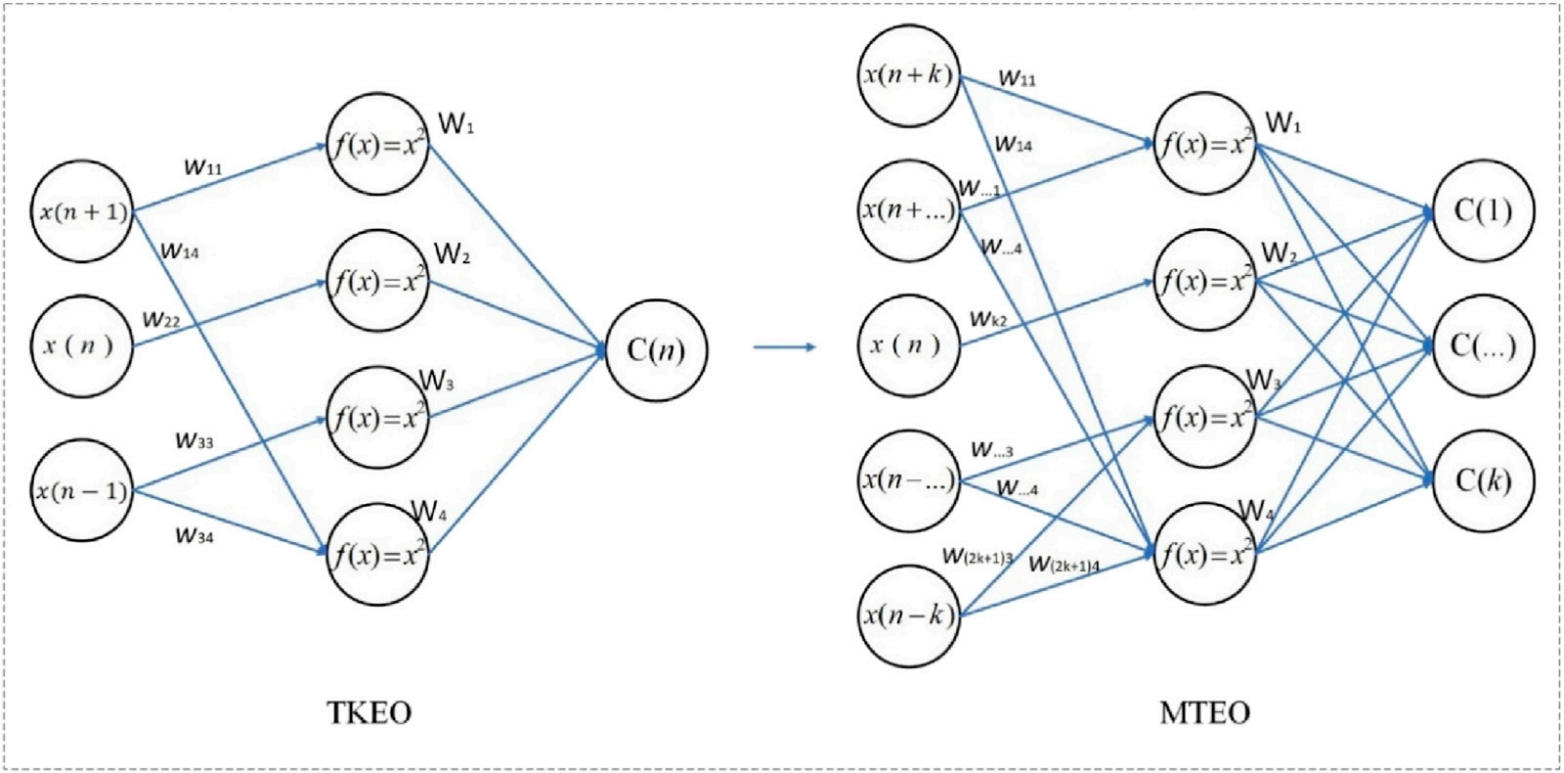
The neural network computational model of energy operators.

If $f\left(x\right)=x^{2}$ is used as the activation function of the convolution layer instead of the commonly used ReLu function, then the neuron connection expression for the energy operator method that outputs the signal “energy” can be obtained as shown in Figure 5. According to Equation 8 and Equation 19, an equivalent formula between the energy operator and the neuron can be established, and based on this relationship, a reasonable neuron parameter configuration scheme can be determined (Equation 20):

$$C\left(k\right)=W_{1}{\left(w_{11}x\left(n+k\right)\right)}^{2}+W_{2}{\left(w_{22}x\left(n\right)\right)}^{2}+W_{3}{\left(w_{33}x\left(n-k\right)\right)}^{2}+W_{4}{\left(w_{14}x\left(n+k\right)+w_{34}x\left(n-k\right)\right)}^{2}=x^{2}\left(n\right)-x\left(n+k\right)x\left(n-k\right)=\Psi_{k}\left[x\left(n\right)\right].$$ (20)

Then, the neuron parameters can be obtained: $W_{1}=W_{2}=W_{3}=1$ , $W_{4}=-1$ , $w_{11}=w_{14}=2$ , $w_{22}=1$ and $w_{33}=w_{34}=\frac{1}{4}$ . By using neurons and weighted connections, the energy operator can process information in parallel with multiple inputs and outputs, as these connections and neurons can be computed concurrently. This exploits the natural advantage of parallel computing inherent in the structure and computation method of the neural network, which enhances the running speed of MEONND. Therefore, by varying k , the convolutional layer with fixed parameters generates multiple TEO sub-sequences that capture the “energy” feature of the EMG signal and apply a high-pass filter to it (Kvedalen, 2003; Wu et al., 2017; Boudraa and Salzenstein, 2018). Subsequently, order statistics can filter out noise interference (Bengacemi et al., 2020) and the pooling layer fuses the features output from the convolutional layer without updating and hyperparameters. The sampling layer replaces the fully connected layer with the most hyperparameters, and normalizes the output of the pooling layer to the [0:1] interval (Equation 21):

$$y_{i}=\frac{x_{i}-\min_{\left(i-2k\right)<i}\left(x_{i}\right)}{\max_{\left(i-2k\right)<i}\left(x_{i}\right)-\min_{\left(i-2k\right)<i}\left(x_{i}\right)}$$ (21)

where i denotes the current sampling point and, the extreme values are obtained on signal segments in the range [ i−2k,i ]. The output of the sampling layer is classified by the activation function, sigmoid, given by Equation 22:

$$f\left(x\right)=\frac{1}{1+e^{-x}}$$ (22)

The sigmoid activation function has two advantages as a classifier: (1) It makes the detector nonlinear; (2) It does not need any prior knowledge about the signal. However, the sigmoid function still has a threshold parameter that needs to be tuned manually, even though it minimizes the number of parameters. In this paper, a hard limiter is used instead of the normal sigmoid function to restrict the output. The hard limiter sets the output value to one when the output of sigmoid exceeds T, indicating muscle activation, and to 0 otherwise, indicating muscle inactivation. Thus, MEONND can return a binary output, 0 or 1, for each sampling point.

**FIGURE 6:**
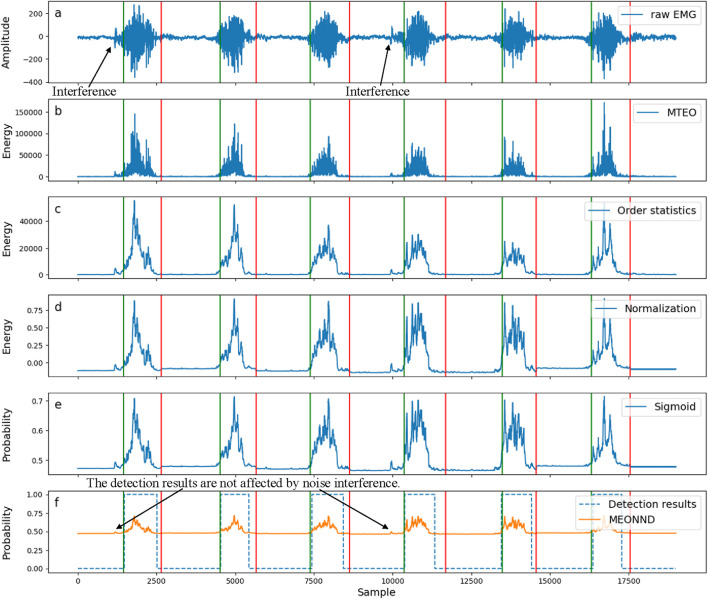
Schematic diagram of the signal changes caused by each functional module when MEONND performs change-point detection on electromyogram (EMG) signals. **(a)** shows the raw EMG signals that have not been processed; **(b–e)** illustrate the effects of the EMG signals after being processed by each layer of MEONND; and **(f)** presents the final result of the change-point detection.

The output of MEONND was post-processed to eliminate the false transitions caused by the stochastic nature of the EMG signal (Ghislieri et al., 2021). The post-processing technique used the intrinsic physiological properties of muscle activity, similar to the threshold method mentioned above. Based on the assumption that the minimum interval between muscle contractions is typically 25–30 m (Yang et al., 2017), any muscle activation shorter than 30 m was discarded (Rashid et al., 2019). The signal changes caused by each module in the MEONND algorithm are shown in Figure 6.

Figure 6 shows the processing of the EMG signal by each functional module of MEONND when detecting change-points. Figure 6a is the raw EMG signal received by the input layer, and the green and red vertical lines are the standard labels of onset and offset points. Figures 6b,c are the results of the processing of the EMG signal by each component of MEONND, and Figure 6f is the result of detecting change-points of the EMG signal by MEONND. The raw EMG signal has obvious noise interference, which is suppressed by the transformation of MEONND, and the classification result is relatively accurate, indicating that the MEONND method has good anti-noise ability and detection accuracy.

### 3.4 Statistical analysis method

An accurate and comprehensive statistical analysis method is important not only for the comparison of algorithm performance but also for the setting of algorithm parameters. The accurate temporal analysis of muscle activation is performed by identifying the burst onset and burst offset to obtain information such as the duration of the activation interval.

The detection results are quantitatively compared against the ground truth in terms of true positive rate (TPR), F1 Score, and onset/offset bias (root mean square deviation, RMSD). More specifically, the indexes are defined as Equations 23, 24, 25:

$$TPR=\frac{N_{detected}}{N_{true}}$$ (23)

$$F1\;Score=\frac{2\times TP}{2\times TP+FN+FP}\times 100$$ (24)

$$Bias=\sqrt{\frac{\sum_{t=1}^{n}{\left({\hat{y}}_{t}-y_{t}\right)}^{2}}{n}}$$ (25)

where $N_{detected}$ represents the number of change-points detected by the algorithms, $N_{true}$ is the correct number of change-points collected by motion data; ${\hat{y}}_{t}$ represents the ground truth of the onset/offset time points and $y_{t}$ is the estimate of the onset/offset time points. The parameters of F1 Score are listed below:

─ TP: prediction result is P, label is P.
─ TN: prediction result is N, label is N.
─ FP: prediction result is P, label Is N.
─ FN: prediction result is N, label is P.

It should be noted that in previous studies, the elements of F1 Score were calculated by treating the active segment as a complete object, or by dividing EMG signals into fixed-length segments (Jubany and Angulo-Barroso, 2016; Rashid et al., 2019; Chopra, 2021). In contrast, this paper investigates the detection performance of onset an offset indiscriminately and therefore requires a redesign of the calculation method for the parameters of F1 Score.

Since the $y_{t}$ detected by the algorithm and the “standard” ${\hat{y}}_{t}$ will hardly be equal, a time region for determining the validity of the detection results needs to be divided according to the location of the ${\hat{y}}_{t}$ . In this paper, the reasonable epsilon interval times are set according to the electrical-mechanical delay of the muscle and the perceivable delay duration of the control system. More specifically, due to the electrical-mechanical delay in voluntary muscle contraction (Vint et al., 2001; Georgoulis et al., 2005), the EMG signal activity can be detected as early as 260 m before the actual movement (Li et al., 2007; Wentink et al., 2013; Yang et al., 2017), and given the latency of the equipment used in this paper, a detection within 300 m before the “standard” change-point is considered valid; furthermore, in practice, the device delay should be less than 200 m to avoid the apparently perceptible system delay (Englehart and Hudgins, 2003). Therefore, this experiment took the motion data as the standard, and the time segments within the range of 300 m before and 200 m after the change point where the motion data was collected were set as the valid detection interval. The valid detection interval divides the EMG signal into four regions: Baseline segment, Onset segment, Activity segment, and Offset segment.

**FIGURE 7:**
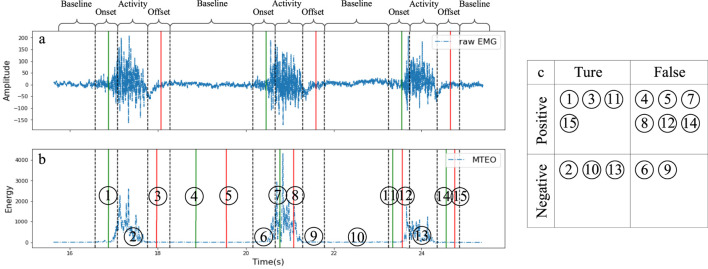
Revised confusion matrix. **(a)** illustrates the four regions of the EMG signal divided by the valid detection intervals and the different classifications of “events” that occur in each region, and **(b)** simulates all possible “events”, **(c)** shows the classification of the “events” detected in **(b)**.

Figure 7a illustrates the four regions of the EMG signal divided by the valid detection intervals and the different classifications of “events” that occur in each region, and Figure 7b simulates all possible “events”, Figure 7c shows the classification of the “events” detected in Figure 7b.:

─ Baseline segment: No events detected count as TN, e.g., No.10; Onset/Offset detected as FP, e.g., No.4 and 5.
─ Activity segment: No events detected count as TN, e.g., No.2 and 13; Onset/Offset detected as FP, e.g., No.7 and 8.
─ Onset segment: Only one Onset detected counts as TP, e.g., No.1 and 11; in other cases, no events detected count as FN, e.g., No.6, multiple events detected or Offset detected count as FP, e.g., No.12.
─ Offset segment: Only one Offset detected counts as TP, e.g., No.3 and 15; in other cases, no events detected count as FN, e.g., No.9, multiple events detected or Onset detected count as FP, e.g., No.14.

The calculation method of F1 Score ensures a balanced sample size and makes the statistical results more reliable. In addition, the statistics are equal for Onset and Offset points.

### 3.5 The parameter tuning model based on the AHP

Currently, the parameters of detectors whether for speech signals (Wu et al., 2017) or biosignals (Rashid et al., 2019; Bengacemi et al., 2020) are usually chosen empirically. However, this paper performs an undifferentiated statistic for onset and offset points, which makes the comprehensive comparison of the five statistical metrics (namely, Onset TPR, Offset TPR, Onset bias, Offset bias and F1 Score) relatively complicated. In addition, the relationship between statistical metrics is not equal, as slightly biased but more robust algorithms are more acceptable for EMG activity monitoring tasks (Tenan et al., 2017). Hence, more important metrics should be given higher weights. This paper introduces the Analytic Hierarchy Process (AHP) to comprehensively consider these five metrics.

**FIGURE 8:**
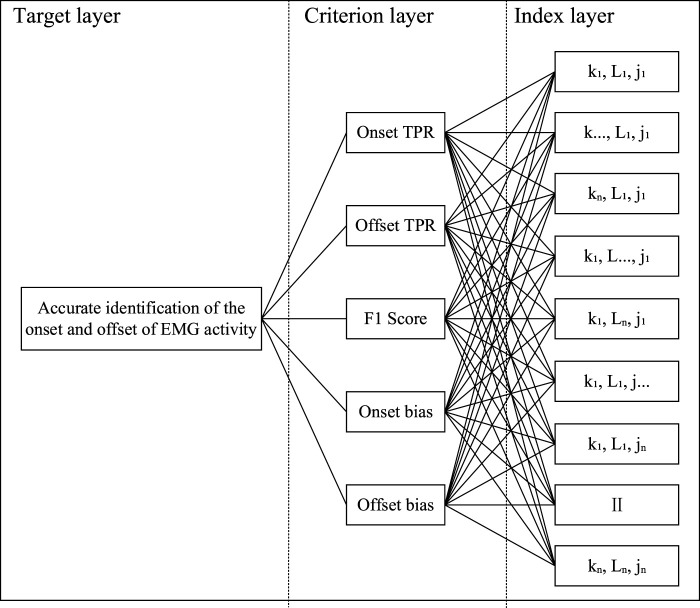
The hierarchical model. The index layer traverses the relationships of all parameters by dint of an exhaustive search.

The AHP is a combination of qualitative and quantitative analysis that allows for a systematic analysis of the impact of the parameters and the internal relationship between them to achieve a comprehensive consideration of statistical metrics (Ho, 2008). To apply the AHP, a hierarchical model must first be developed based on the purpose of the experiment. The hierarchical model designed in this paper is shown in Figure 8. The AHP process visualizes the decision-making process through the hierarchical model. As a bottom-up evaluation model, higher levels of the hierarchy are influenced by lower levels (Forman and Gass, 2001). Therefore, to achieve the target layer, a comparison of the complex relationships between the factors in the criterion layer and the index layer needs to be addressed. AHP realizes pairwise comparison among the factors by establishing a judgment matrix and weight division to decompose a complex problem step by step. Firstly, this paper takes the 1–5 scaling method to construct the judgment matrix. The consistency test (CI = 0.0033, RI = 1.11, CR = 0.003 < 0.1) proves that the weights assigned to each factor are reasonable. Subsequently, the maximum eigenvalue and eigenvector are calculated. Finally, the weight vector of the single hierarchical order representing the influence degree of the underlying factors for the upper is calculated. The detailed parameter settings and calculation results are shown in Table 1.

**TABLE 1:** The judgment matrix, eigenvector and weight set.

	Onset TPR	Offset TPR	F1 Score	Onset bias	Offset bias	Feature vector	Weight set
Onset TPR	1	1	2	3	3	1.7826	0.3134
Offset TPR	1	1	2	3	3	1.7826	0.3134
F1 Score	0.5	0.5	1	2	2	1	0.1758
Onset bias	0.3333	0.3333	0.5	1	1	0.561	0.0986
Offset bias	0.3333	0.3333	0.5	1	1	0.561	0.0986

Max-Eigen = 5.0133, CI = 0.0033, RI = 1.11, CR = 0.003 < 0.1.

It should be noted that the creation of a judgment matrix generally requires material support (e.g., questionnaires). Due to the specificity of the experiments in this paper, the judgment matrix can only be set subjectively by experts. Therefore, The AHP process can only achieve semi-subjective parameter tuning. As mentioned above, a slightly biased but highly reliable algorithm is more acceptable for EMG activity monitoring tasks (Tenan et al., 2017). With that bias in mind, the Onset/Offset TPR and F1 Score have a higher priority compared to the Onset/Offset bias. In addition, in the comparison of Onset/Offset TPR and F1 Score, the strategy of removing as many negative samples as possible while ensuring positive samples is more reasonable and acceptable. Therefore, Onset/Offset TPR should be assigned more weights than F1 Score. The judgment matrix shown in Table 1, which was constructed based on the above view, was consistent with the subjective judgment of the experts.

### 3.6 The reasonable parameter traversal intervals

The MEOTD and MEONND monitors have three parameters that need to be manually optimized: the scale factor k for MTEO; the window length L required for the order statistics; and the scale factor j/T for the threshold. The prerequisite for parameter optimization by the AHP model is to give a reasonable traversal interval for the parameters to be tuned, respectively. It should be particularly noted that the MEONND model designed in this paper draws on the organizational structure of the convolutional neural network. However, in contrast to the convolutional neural network, the “neuron” parameters of the MEONND model are preset according to the calculation rules of the energy operator. Essentially, this model is driven by mathematical rules rather than data. Therefore, the method for updating the model parameters in this study is completely different from the method for updating the internal parameters of traditional neural networks. Considering that the remaining three adjustable parameters of the MEONND are similar to the hyperparameters in traditional neural network algorithms, they can be matched and set according to the recommended range.

**FIGURE 9:**
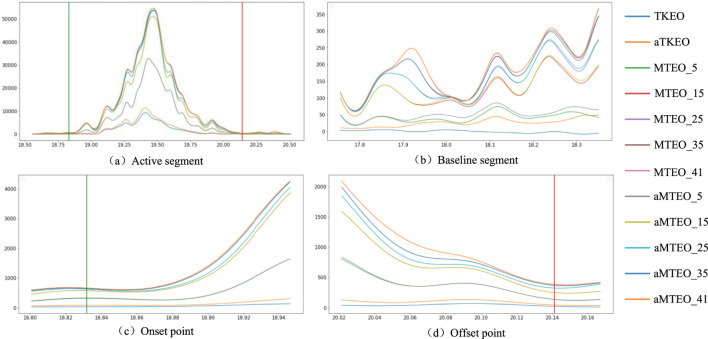
Schematic diagram of the envelope lines of a randomEMG active segment with different resolution parameters k. The green and red vertical lines are the “standard” onset point and offset point of the EMG activity, respectively. In the legend, the text before and after the underline identify the parameter configuration of the algorithm. **(a,b)** represent the active segment and the resting segment respectively, while **(c,d)** represent the onset point and the offset point respectively. For the sake of clear demonstration, the y-axis has been scaled to achieve the best presentation effect.

In general, the values of k used in the MTEO are in the range 1 to 20 , which covers the sampling frequencies of 10–40 kHz in practical neural signal recording systems (Choi and Kim, 2002). The linear envelopes of a random EMG active segment are obtained for MTEO and aMTEO by a second-order Butterworth filter with zero lag for visualization. Figure 9 clearly demonstrates the envelope variation of the MTEO and aMTEO processed signals, starting from k = 1 (i.e., the conventional TKEO) to 41, with an interval of 5. According to Figure 9, two assumptions can be raised: (1). After the resolution factor k exceeds 25, the effect on the detector performance will reach saturation; (2). The effect of full-wave rectification on MTEO is negligible.

To verify the above assumptions, in this experiment, k is considered to be an odd number within 1 - 41 , which ensures that the frequency range of the normal EMG signal is covered (Choi and Kim, 2002; Wu et al., 2017; Boudraa and Salzenstein, 2018). Since the relationship between L and k is L=2k+1 (e.g., when k=15 , each calculation involves a symmetric time-window centered on the sample point, for a total of 31 samples), it is reasonable that the interval of L is set to an odd number within 3–83. In addition, the threshold scale factor j for the MEOTD is set to 1–10, a range of values that possess statistical significance and has been used in many previous studies (Solnik et al., 2008; Wu et al., 2017). The threshold parameter T of the MEONND is determined by the sample distribution. In general, if the sample distribution is absolutely average, the decision boundary of sigmoid is 0.5. However, bias is inevitable in the experimental data, although the statistical method used in this paper, as shown in Figure 7, has attempted to maintain the average of the sample distribution as much as possible. Therefore, the traversal range of the threshold T is set to [0.406:0.514] around 0.5 with an interval of 0.02.

## 4 Results

The dataset of each participant was divided into a training set (40%) and a test set (60%). Thus, a total of 2,880 training samples and 4,320 test samples were available. First, the results supporting that MTEO has better signal conditioning than TKEO are shown by the AHP parameter tuning model; and, the effect of full-wave rectification on the energy operator approaches. Second, the effectiveness of the method in this paper is further verified by comparing the state-of-the-art monitors.

### 4.1 Signal conditioning performance comparison

Introducing the AHP model into the experiments in this paper requires adjusting the computational rules. Since the Onset/Offset bias is not uniform with the other three statistical metrics units and has an inconsistent direction of influence on the algorithm (the larger the deviation the lower the performance). Therefore, the strategy of this paper is to control the impact of Onset/Offset bias on the AHP model to three and 4 decimal places and take negative values. The details are as Equation 26:

$$AHP=Onset\;TPR*w_{1}+Offset\;TPR*w_{2}+F1\;Score*w_{3}-Onset\;bias/100000*w_{4}-Offset\;bias/100000*w_{5}$$ (26)

where /100000 is to control the impact range of the Onset/Offset bias metrics, and −Onset/Offset bias is to adjust the impact direction. Table 2 shows the best combination of parameters obtained by the AHP model on the training set.

**TABLE 2:** Parameter combinations for the maximum AHP obtained by the monitors and statistical results representing the performance of the monitors.

Detector	k	L	j/T	Onset TPR (%)	Offset TPR (%)	F1 Score (%)	Onset bias (ms)	Offset bias (ms)	AHP
TEOTD	─	47	9	85.17 ± 8.75	77.67 ± 13.24	82.53 ± 6.32	183 ± 78	295 ± 93	0.6550
aTEOTD	─	31	7	90.23 ± 6.91	88.87 ± 9.33	88.29 ± 4.49	154 ± 66	255 ± 87	0.7161
MEOTD	15	15	5	95.15 ± 5.43	92.27 ± 7.74	91.67 ± 3.77	133 ± 58	239 ± 73	0.7482
aMEOTD	15	15	4	95.16 ± 5.59	92.83 ± 6.96	91.80 ± 3.68	117 ± 53	223 ± 72	0.7502
MEONND	15	15	0.506	97.31 ± 3.53	93.33 ± 6.11	93.48 ± 3.55	103 ± 21	209 ± 71	0.7615
aMEONND	15	15	0.506	97.31 ± 3.53	93.58 ± 6.00	93.72 ± 3.51	102 ± 19	204 ± 54	0.7627

TD, and NND, are used to distinguish detectors, while TEO, and MEO, are used to distinguish energy operators, and ‘a’ denotes full-wave rectification.

According to Table 2, three points can be drawn: (1). The MTEO has better signal conditioning than the TKEO, which makes the performance of the threshold detector more stable; (2). Full-wave rectification contributes significantly to the performance improvement of the TKEO, but the effect on the MTEO is negligible; (3). The monitors detect onset points more accurately than offset points. Based on the above findings, only MEOTD and MEONND are required to be compared with other monitors to highlight the performance of the algorithm in this paper under the condition of optimal combination of parameters (MEOTD with k=15 , L=15 , j=5 , and MEONND with k=15 , L=15 , T=0.506 ), while the conventional aTEOTD is retained as a performance reference.

### 4.2 Performance comparison of EMG activity monitors

To highlight the detection performance of the monitor constructed in this paper, we compare it with the state-of-the-art monitors and present the sources and parameter settings for all monitors in Table 3.

**TABLE 3:** Parameter configuration of the monitors used for comparison.

Monitor	Rectification or filtering	Windowing parameter	Threshold factor	Special parameters	Notes
aTEOTD	Yes	31	7	N/A	
MEOTD	No	15	7	k = 15	
MEONND	No	15	0.506	k = 15	
Sample Entropy (SE)	No	L = 32, S = 4	0.6	m = 2 r = 0.25 × SD	Zhang and Zhou (2012)
Bayesian Changepoint Analysis (BCA)	Yes	N/A	N/A	ω = 0.2 p = 0.0 95% CI	Tenan et al. (2017)
Profile Likelihood Maximization-Log normal (PLM-Log)	Yes	N/A	N/A	N/A	Selvan et al. (2018)
Long short-term memory recurrent neural network (LSTM)	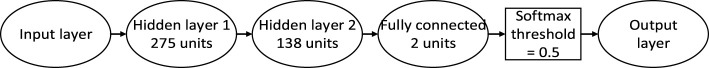				Ghislieri et al. (2021)

Table 3 shows that the Profile Likelihood Maximization method (Selvan et al., 2018) proposed by Selvan et al. requires the least number of parameters. However, they have tested various probability distributions and probability density functions before drawing their conclusions, which is a significant amount of work. Moreover, PLM-Log uses TKEO to pre-process the signal. The Bayesian Changepoint Analysis method (Tenan et al., 2017) tested by Tenan et al. does not require a moving window function or a threshold factor, but it does require traversing the posterior probability within [0.60, 0.95] with a fixed parameter. The Sample Entropy (SE) algorithm (Zhang and Zhou, 2012) proposed by Zhang et al. is similar to the threshold algorithm in that it requires iteration to obtain a reliable threshold factor. Pattern recognition algorithms based on neural networks are generally used for myoelectric control, hand gesture or upper limb movement recognition, and motion intent detection (Yousefi and Hamilton-Wright, 2014; Shim et al., 2016; Phinyomark and Scheme, 2018). Indeed, the neural network algorithm still performs well in one-dimensional time-series tasks (Akef Khowailed and Abotabl, 2019; Ghislieri et al., 2021). Nevertheless, the initialization settings of parameters such as the number of hidden layers, number of hidden units of each hidden layer, learning rate values, and drop period values have a decisive impact on the algorithm performance. Moreover, the neural network architecture design depends on the training data set. All these issues complicate the use of neural network algorithms for EMG activity monitoring. The Long short-term memory recurrent neural network (LSTM) (Ghislieri et al., 2021) proposed by Ghislieri et al. has two LSTM layers with 275 units in the first layer and 138 in the second; according to the number of classes to be recognized, the fully connected layer is set to two units; and the threshold of the activation function sigmoid is set to 0.5. The algorithm structure is detailed in Table 3.

**FIGURE 10:**
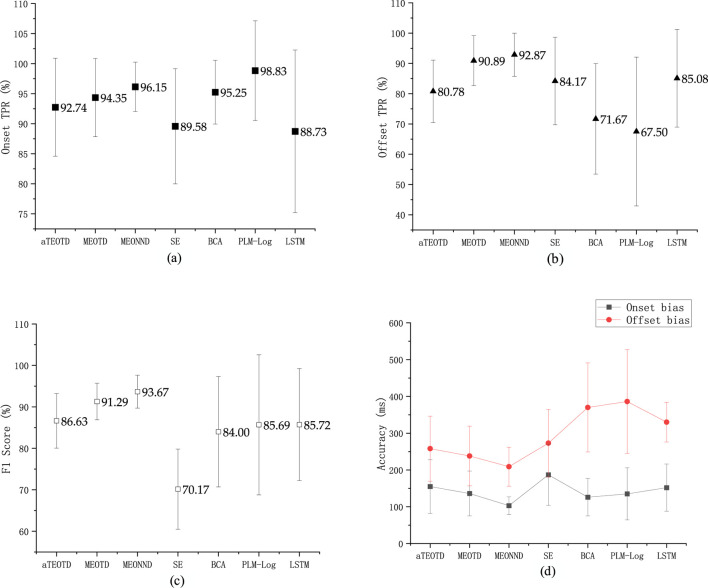
Values of **(a)** Onset TPR, **(b)** Offset TPR, **(c)** F1 Score and **(d)** Accuracy, averaged on the test set. Error bars represent the standard errors.

We present the results obtained from the test set for the seven different approaches in Table 4 and Figure 10.

─ Onset TPR: The average Onset TPR on the test set was 92.74% ± 8.15%, 94.35% ± 6.51%, 96.15% ± 4.11%, 89.58% ± 9.59%, 95.25% ± 5.31%, 98.83% ± 8.31% and 88.73% ± 13.53% for the seven monitors, respectively. The PLM method achieved the best performance in this metric, but it was less stable than the method in this paper, as indicated by the larger standard deviation.
─ Offset TPR: In contrast to the Onset TPR, all monitors exhibited some degree of degradation in detection performance for the Offset points. Only two monitors exceeded 90%, namely, MEONND (92.87%) and MEOTD (90.89%).
─ F1 Score: Due to the low Offset TPR metrics, the F1 Score metrics of all monitors were generally low. The method proposed in this paper achieved the highest F1 Score, where MEONND was the best (93.67% ± 3.97%), followed by MEOTD (91.29% ± 4.41%). F1 Score is a comprehensive metric used in statistics to measure binary classification models, which takes into account both the true detection rate and the false detection rate. Thus, although showing consistent performance trends with the TPR metrics, the F1 Score metric can reveal some additional characteristics. For example, the Onset TPR and Offset TPR of SE are both much higher than the F1 Score, indicating that SE has a more serious false detection problem.
─ Onset bias: The average Onset bias metric on the test set was 155 ± 73 m, 136 ± 61 m, 103 ± 24 m, 187 ± 83 m, 126 ± 51 m, 135 ± 71 m, and 152 ± 64 m for the tested muscle activity detectors, respectively. MEONND had the smallest bias and BCA was the second smallest.
─ Offset bias: The Offset bias metrics were larger than the Onset bias metrics for all algorithms, which is consistent with the experimental expectation based on the discharge characteristics of MUAPs (Kang et al., 2020; Wang et al., 2022).

**TABLE 4:** Parameter combinations for the maximum AHP obtained by the monitors and statistical results representing the performance of the monitors.

Detector	Onset TPR (%)	Offset TPR (%)	F1 Score (%)	Onset bias (ms)	Offset bias (ms)
aTEOTD	90. 74 ± 8.15	87.78 ± 10.33	86.63 ± 6.59	155 ± 73	258 ± 88
MEOTD	94.35 ± 6.51	90.89 ± 8.21	91.29 ± 4.41	136 ± 61	238 ± 81
MEONND	96.15 ± 4.11	92.87 ± 7.14	93.67 ± 3.97	103 ± 24	209 ± 53
SE	89.58 ± 9.59	84.17 ± 14.46	70.17 ± 9.68	187 ± 83	273 ± 92
BCA	95.25 ± 5.31	71.67 ± 18.25	84.00 ± 13.33	126 ± 51	370 ± 121
PLM-Log	98.83 ± 8.31	67.50 ± 24.58	85.69 ± 16.89	135 ± 71	386 ± 141
LSTM	88.73 ± 13.53	85.08 ± 16.11	85.72 ± 13.51	152 ± 64	330 ± 54

### 4.3 Comparison of running time

Running time is an important consideration for EMG activity monitoring algorithms, especially in real-time applications. We implemented all algorithms using Python 3.8.5 on a computer with an Intel Core i5-3210M CPU (2.50 GHz) and 8.00 GB of RAM. Table 5 shows the average running times for one data acquisition from ten random participants.

**TABLE 5:** Running time comparisons.

	aTEOTD	MEOTD	MEONND	SE	BCA	PLM-log	LSTM
Total (ms)	56.1 ± 4.31	139.2 ± 6.06	57.6 ± 4.87	2,762.3 ± 89.11	550.8 ± 13.28	619.5 ± 14.31	265.5 ± 10.95
Average (ms)	1.87	4.64	1.92	92.07	18.36	20.65	8.85

As Table 5 shows, the total and average running times represent the total time to detect 30 voluntary muscle contractions and the average time to detect one voluntary contraction, respectively. The adaptive Teager energy operator and double-threshold (aTEOTD) method is the fastest performing method, because it consists of two fast algorithms: the TKEO and the double-threshold method. The sample entropy (SE) method is the slowest, so it is generally not recommended to combine the energy operator algorithms with the SE method because the advantages of the energy operator cannot be exploited. Another advantage of the proposed method is that the modified energy operator and nearest neighbor distance (MEONND) method is significantly faster than the modified energy operator and double-threshold (MEOTD) method and even close to the aTEOTD method, indicating that the second monitor construction strategy proposed in this paper is more advantageous in terms of execution efficiency. Finally, comparing the long short-term memory (LSTM) and MEONND methods, it can be demonstrated that the modified Teager energy operator (MTEO) requires more computation than the LSTM layers for processing signal sampling points.

## 5 Discussion

The experimental objectives of this paper can be summarized as follows: (1) to verify whether this variant of the TKEO, MTEO, can effectively perform the muscle activity detection tasks; (2) to determine whether full-wave rectification is necessary; (3) to construct an effective parameter tuning model; and (4) to verify whether the monitor construction strategy proposed in this paper is reliable..

**FIGURE 11:**
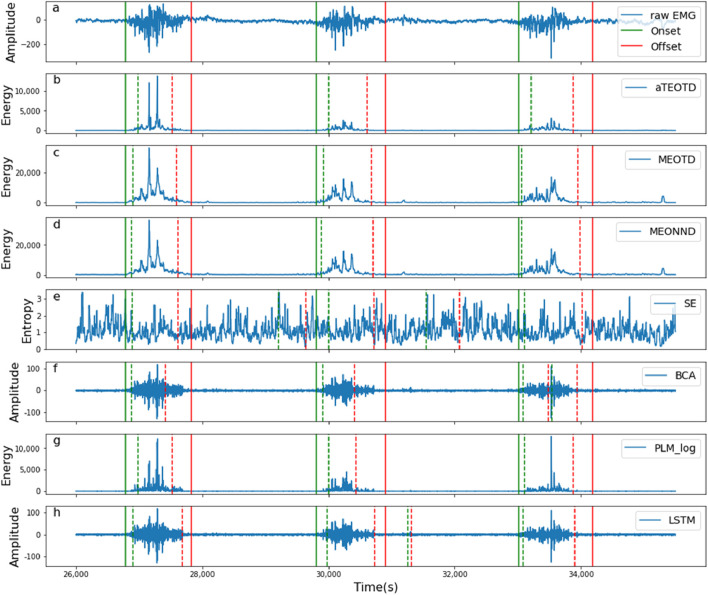
Schematic diagram of random segments of seven monitors for change-point detection of EMG signals. **(a)** shows the raw EMG signal and the ground truth values of the change points. **(b–h)** show the EMG signal processed by the monitor (featured by ‘energy’, ‘amplitude’ and ‘entropy’) and the change points of the signal detected by the monitors.

─ *The superiority of the MTEO.* Previous studies have shown that preprocessing with various versions of energy operators, such as ETKEO (Tigrini et al., 2020), EGTKO (Jabloun, 2017), and MTEO (Wang et al., 2022), can enhance the performance of myoelectric signal change point detection algorithms, compared to the conventional TKEO operator. The MTEO has a stronger signal conditioning capability than multiple TKEO variants (Wang et al., 2023), because it combines multiple energy operator subsequences using pooling techniques, which capture more signal frequencies than a single energy operator sequence, thereby increasing the SNR and reducing the noise variance (Choi and Kim, 2002; Tigrini et al., 2020). Moreover, the pooling layer helps to aggregate semantically similar features into one, and to make the representations more invariant to variations in position and appearance of the elements in the previous layer (LeCun et al., 2015). Thus, the MTEO technique with a pooling layer has a more robust signal conditioning capability. The experimental results confirm that using the MTEO and TKEO in the preprocessing step of the same threshold detector can reveal the performance difference between them. As shown in Table 4, the MEOTD outperforms the TEOTD in terms of Onset TPR, Offset TPR, F1 Score, Onset bias and Offset bias by 3.98%, 3.54%, 5.38%, 12.26% and 7.75%, respectively. Therefore, the MTEO is more effective for improving detector performance.
─ *The impact of full-wave rectification on the energy operators.* The full-wave rectification enhances the performance of the conventional TKEO operator significantly, but has a negligible effect on the MTEO. As shown in Table 2, for the five performance metrics, the aTEOTD improved by 5.94%, 14.42%, 6.98%, 15.85% and 13.56%, respectively, compared to the TEOTD. However, the performance difference between the two monitors using MTEO was less than 0.6% for all metrics. Therefore, the impact of full-wave rectification on the MTEO can be considered negligible in practice. As explained above, the MTEO employs pooling technique, and the pooling layer makes the representations more invariant to changes in the previous layer. Moreover, full-wave rectification may cause signal distortion (Negro et al., 2015). Therefore, the MTEO does not require full-wave rectification of the signal, which is an advantage over the conventional TKEO.
─ *The parameter tuning model based on the AHP.* The parameter tuning model based on the AHP. Although the strategies proposed in this paper for building muscle activity monitors based on MTEO have reduced the number of manually tuned parameters, there are still two to three key parameters that influence performance. The threshold detector-based approach requires the combination of three parameters, while the neural network architecture-based detector requires only two parameters to balance the detection performance, as it does not need the extraction of baseline segment prior knowledge. However, even tuning only two parameters is a relatively complex problem. Typically, after fixing one parameter empirically, the optimal value for the other parameter is searched by an exhaustive method. But this way of setting one parameter based on experience is too subjective, and the effect of each parameter on detector performance is not synchronized, so how to balance multiple parameters is a problem that needs to be solved. The AHP can objectively calculate the optimal combination of parameters after providing the weight of each parameter. The AHP method is a semi-subjective approach, as it requires the prior assignment of different weights based on the importance of the input elements. Although the subjective part cannot be avoided, the computational process that follows the subjective part is completely objective and can distinguish subtle performance differences and assist in the decision-making process. Therefore, the parameter tuning model developed in this paper can traverse the parameters in a relatively dense manner to select a more accurate and optimal combination of parameters.
─ *The effectiveness of the monitor construction strategies.* This paper proposed two strategies for building muscle activity monitors based on the MTEO. One is the common approach based on a threshold detector, and the other is a strategy to construct a detector by replacing part of the functional components of the CNN. By comparing the two construction strategies with the state-of-the-art monitors, the effectiveness of the MTEO for muscle activity detection was demonstrated on one hand, and the improvement of the monitors developed in this paper was verified on the other hand. The usual strategy for muscle activity monitors based on the conventional energy operator (TKEO) is to incorporate a threshold detector (Xiaoyan and Aruin, 2005; Li et al., 2007; Solnik et al., 2008; 2010; Rashid et al., 2019; Bengacemi et al., 2020; Tigrini et al., 2020). TKEO has been used in the pre-processing phase of many change-point detection algorithms, but this variant of TKEO, MTEO, has rarely been explored in muscle activity detection tasks, although it has been shown to have significant value in speech applications (Wu et al., 2017). Table 4, 5 show the detection performance of the two monitors proposed based on the MTEO. Although the first construction strategy is relatively more conventional, it still has good detection performance and running speed. The new alternative construction strategy proposed in this paper further improves the detection performance of the algorithm, especially in terms of running speed which is already comparable to the scheme combining the TKEO and a threshold detector.
─ *The reliability of the statistical method for representing the algorithm performance.* The main purpose of the change-point detection algorithm for EMG signals is to extract temporal information, such as the onset time point, duration and offset time point of muscle activity. When EMG signals are used as control signals or for clinical analysis, it is important to accurately extract the complete temporal information. Unlike previous studies that only examined the detection performance of onset points (Tenan et al., 2017; Selvan et al., 2018), this paper assigned the same weight to onset point and offset point for evaluation. Considering that the detection accuracy would not match the gold standard, four valid detection intervals were defined to classify the detection results, as shown in Figure 7. Depending on the valid detection interval where the detected change-points were located, they can be classified into three categories: false detections, missed detections and correct detections. Figure 11 illustrates a random segment of the detection results of all monitors involved in the comparison. Taking Figure 11a as a reference, the monitors show different degrees of bias in detecting muscle activities. For example, Figure 11f shows the detection results of the BCA monitor. Although the BCA detected a part of the active fragment of the EMG signal, the detection of the offset point was outside the valid detection interval and therefore could not be considered as a valid detection result. Similarly, as shown in Figure 11g, the PLM_log monitor also resulted in lower performance metrics because of this reason.
─ *The generalization capability of the model.* This study conducted a thorough evaluation of the generalization capability of the proposed monitor. It is particularly noted that the assessment of the model was based solely on using 40% of each subject’s data as the training set and the remaining 60% as the test set. While this analytical method can assess the model’s adaptability to individual physiological signals, it does not fully demonstrate its capacity to handle new data. To more accurately evaluate the model’s generalization ability, this study supplemented the assessment with cross-validation results, randomly selecting 40% of the subjects’ data for training and the remaining 60% for testing, to more authentically reflect the monitor’s response to unknown data. The experimental results, with an average accuracy of 74.34% ± 8.06%, indicate that although parameter adjustment through the Analytic Hierarchy Process (AHP) achieves good performance on known data, the model’s generalization capability on unseen data remains limited. This limitation is mainly due to two factors: firstly, the high randomness of human physiological data, for which there is currently no effective solution; secondly, the proposed strategy focuses on detection speed and personalized application scenarios, and the research is still in its preliminary stages. Although the test results for the model’s generalization performance are not ideal, considering the specificity of human physiological data and the specialized nature of most application scenarios, the method proposed in this paper still has practical application value. In the future, to further enhance the model’s generalization ability, research will be devoted to introducing more advanced feature learning models to improve and optimize the existing methods.

## 6 Conclusion

This paper tackles a long-standing and relatively straightforward problem, namely, the detection of the change-points of EMG signal activity. Based on a signal pre-processing technique, MTEO, which has a good performance in the field of speech signal processing, this paper proposes two strategies to effectively construct muscle activity monitors. The MEOTD is based on a threshold method detector that has been validated several times by the conventional TKEO (Li et al., 2007; Solnik et al., 2010; Rashid et al., 2019; Bengacemi et al., 2020); while the MEONND replaces the components that need to be trained in the convolutional neural network architecture with components that have been validated as effective for the same role. The principle of both strategies is intuitive. The signal-to-noise ratio (SNR) of the EMG signal is effectively improved by the MTEO, and the ‘energy’ features of each sampling point are acquired. When the energy of the sampling point exceeds the pre-set threshold and accumulates to a certain amount, it is determined as the onset point of muscle activity. A similar process can obtain the offset point of muscle activity. This strategy relies heavily on the signal features of the baseline segment used to estimate the threshold. This paper provides an alternative scheme to address the drawback that the threshold detector requires prior knowledge about the signal baseline segment. Not only does the nonlinear classifier in the CNN architecture enhance the robustness of the algorithm, but also the real-time performance of the neural network is fully utilized to improve the efficiency of the muscle activity detection algorithm. Compared with state-of-the-art monitors, the monitors constructed in this paper are not only robust and accurate, but also have very high detection efficiency.

Future research needs to explore the change-point analysis performance of MEOTD and MEONND in more complex EMG waveforms to determine their feasibility in more complex tasks. Moreover, updating the semi-subjective parameter tuning model to enhance the self-updating capability of the monitors.

## Data Availability

The raw data supporting the conclusions of this article will be made available by the authors, without undue reservation.
